# Soil and vegetation property data from the Ultuna R3-RAM56 long-term soil amendment experiment, 1956–2023

**DOI:** 10.1016/j.dib.2025.111350

**Published:** 2025-01-30

**Authors:** Grace Pold, Emme MacDonald, Sabina Braun, Anke M. Herrmann

**Affiliations:** Department of Soil and Environment, Swedish University of Agricultural Sciences, Box 7014, 750 07 Uppsala, Sweden

**Keywords:** Soil carbon, Crop yields, Long-term experiments, Fertilization, Agriculture

## Abstract

The R3-RAM56 long-term experiment in Ultuna, Sweden has tracked the effect of organic and inorganic amendments on agricultural crop and soil properties for the past almost 70 years. Aboveground crop biomass has been collected annually since the beginning of the experiment, while topsoil samples were collected three times in the first twenty years and every other year since 1963. We report crop yields and nutrient content, as well as carbon, nitrogen, and micronutrient content for soils and amendments. The data presented here provides the backbone for numerous published studies in international peer-reviewed journals, and provides a useful time series for calibrating models of soil carbon and nitrogen stocks under varying agricultural management practices in relation to soil amendments.

Specifications Table

**Table:** 

Subject	Agricultural Sciences
Specific subject area	Soil science, soil nutrient cycling, and agricultural management practices
Type of data	Raw tables
Data collection	Soil samples were collected to 20cm at the onset of the experiment, at 9, 18 and 19 years after the onset of the experiment, and every two years thereafter. All aboveground crop biomass was collected at the end of each growing season and split into agronomically-relevant fractions. Soil amendment nutrient and dry mass content are also reported. All samples were analyzed using standard soil and plant testing protocols at the time of collection. Historical datasets were compiled and digitized to generate this dataset, and units were harmonized. Data lacking units, corresponding to biologically-infeasible values, or for variables collected only once over the course of the experiment were excluded.
Data source location	The data were collected from Ultuna, Sweden (59.81256, -17.65095). Archived soil and plant samples and original reported data and field notes are stored in the Department of Soil and Environment, Swedish University of Agricultural Sciences, Ultuna.
Data accessibility	Repository name: Swedish National Data Service Data identification number: SND-ID: 2024-468 Dataset DOI: https://doi.org/10.5878/7f5n-vp94 Direct URL to data: https://snd.se/en/catalogue/dataset/2024-468
Related research article	None

## 1 Value of the Data

• This dataset synthesizes almost 70 years of soil chemistry, plant production, and plant nutritional content data from one of the best-studied long-term agricultural field experiments in Sweden, putting it in its entirety into the public sphere for the first time.
• Longitudinal and replicated nature of dataset makes it useful for evaluating changes in soil properties and plant production under different soil amendment regimes.
• Data can be used to revise models of soil carbon stocks under different proposed land use management practices alone or in comparison to similar experiments run elsewhere.

## 2 Background

Addressing the dual problems of diminishing soil carbon stocks and the push for greater crop yield requires a fresh look at the variety of soil amendments available. The dataset described here is derived from an experimental site established in 1956 to compare the effects of different organic matter sources on agricultural productivity and soil fertility. Results from this field site have been used extensively since it was established, including to evaluate a physical model of soil organic matter turnover [1], study the fate of organic pollutants [2], and assess the effects of stress on microbial activity [3,4]. However, much of the historical baseline data from this experiment had not been digitized or made available in full in public repositories, which we address with the dataset presented here.

## 3 Data Description

**Fig. 1:**
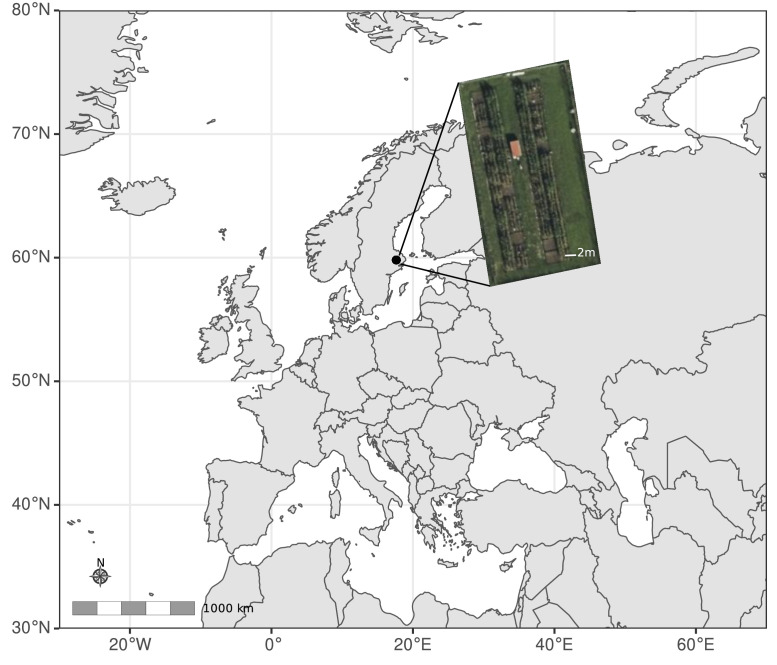
Map showing location and plot layout of the RAM56 experimental site in Ultuna, Sweden. Map generated using the sf [8], ggplot2 [9], ggspatial [10], and rnaturalearth [11] packages in R [12]. Aerial view of plots in inset from GoogleEarth.

**Fig. 2:**
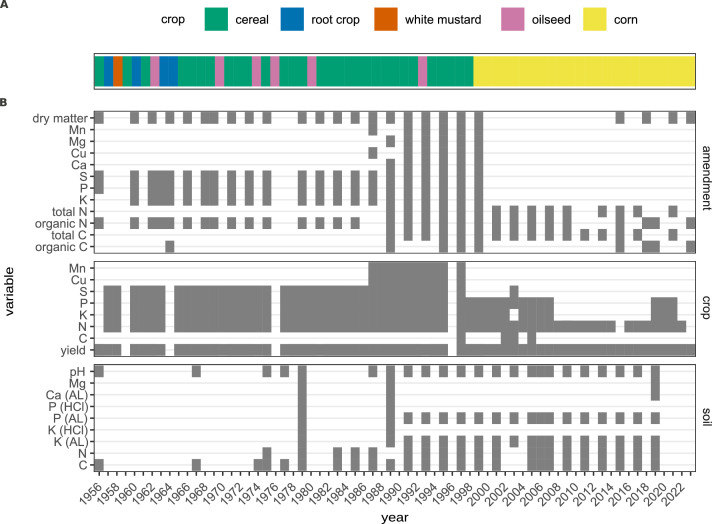
Timeline of crop type (a) and availability of data by year and compartment in dataset described here (b). The coloured bar in a. corresponds to the crop type planted that year. In b. “amendment” corresponds to the material added to soil; “crop” to any crop-derived variables; and “soil” to any soil-derived variables. “AL” in variable names denotes ammonium-lactate extractable nutrient, while HCl denotes hydrochloric acid extractable nutrients. No data are available for 1959 due to crop failure, and in 1976 due to uneven bird damage.

The data described in this article are derived from a long-term soil amendment study initiated in 1956 in Ultuna, Sweden (Fig. 1; [5]). This study was established to determine how different combinations of organic matter additions and nitrogen fertilizer affect soil organic matter stocks, crop production, and plant and soil nutrient pools. In the first year following plot establishment, oats were planted to check homogeneous productivity over the experimental area [5]. Subsequently, each 2 × 2 m plot has received one of fifteen treatments (Table 1), with four replicates per treatment. The plots are arranged in order of expected productivity in the first block, while the remaining three blocks follow a randomized block design. Organic amendments were added in autumn of 1956, 1960, 1963 and every second year thereafter. These amendments are applied after harvest and the removal of any residual aboveground material and worked into the soil to a depth of 20cm [6]. Amendment rates are 8000 kg dry matter ha^-1^ application^-1^ (1956 and 1960) or 8000 kg ash-free organic matter ha^-1^ application^-1^ (1963 onwards), with an average of 50 % C in organic matter assumed in calculations [6]. Inorganic nitrogen fertilizer, if used, is added yearly in the spring [7]. All treatments except treatment K receive 20 kg P and 35–38 kg K ha^-1^ yr^-1^, which has been added in various forms in spring since the experiment was initiated. Plots were planted with alternating cereals, oil seed, or root crops until 2000, when solely forage maize (*Zea mays*) started to be grown (Fig. 2a). All aboveground biomass is removed and recorded each year, while belowground biomass is reincorporated into the soil. Topsoil samples were collected four times in the first two decades of the experiment and approximately every other year since (Fig. 2b). All management of the plots is done by hand, including tilling, sowing, fertilization, organic matter amendment and harvesting. Originally 40cm deep wood and subsequently metal frames were dug approximately 30cm into the soil to minimize transfer of soil between plots. This experiment is also listed in Global Long-Term Agricultural Experiment Network (GLTEN; https://glten.org/experiments/324) and BONARES (https://tools.bonares.de/ltfe/lte-details/85/). The experiment is ongoing.

**Table 1:** Description of treatments included in this study.

Treatment code	Organic material	Nitrogen
A	No organic material; bare fallow	None
B	No organic material	None
C	No organic material	80 kg ha^-1^ as calcium nitrate
D	No organic material	80 kg ha^-1^ as ammonium sulphate
E	No organic material	80 kg ha^-1^ as calcium cyanamide
	Straw	None
G	Straw	80 kg ha^-1^ as calcium nitrate
H	Green manure (grass; no leguminous material)	None
I	Sphagnum peat	None
J	Manure (solid cattle)	None
K	Manure (solid cattle) with superphosphate	None
L	Sawdust (pine or spruce)	None
M	Peat	80 kg ha^-1^ as calcium nitrate
N	Sawdust (pine or spruce)	80 kg ha^-1^ as calcium nitrate
O	Anaerobically digested sewage sludge	None

Topsoil samples are collected prior to amendment addition in the autumn and evaluated for percent carbon and nitrogen by mass, pH and extractable nutrients. Plant samples are evaluated for harvest yield, macro- and micronutrient content (Fig. 2b). Organic soil amendments are similarly evaluated for dry matter and nutrient content, including nitrogen, phosphorus, potassium and sulfur, heavy metals, and organic matter or carbon content.

The dataset contains 7 tab-delimited text files and one svg file described in Table 2.

**Table 2:** Description of files included in this dataset.

Table	Title	Parameters in table
0	RAM56_data.tsv	Plant, soil and amendment data as described in README and Fig. 2
1	RAM56_README.txt	Descriptors for each variable presented in subsequent tables
2	RAM56component_descriptions.tsv	Definitions of data components listed
3	RAM56plot_metadata.tsv	Definitions of plot treatments
4	RAM56unit_descriptions.tesv	Definitions of measurement units
5	RAM56variable_descriptions.tsv	Definitions of variables listed
6	RAM56_harvest_dates.tsv	Dates of crop harvest
7	RAM56plot_metadata.svg	Plot layout map

## 4 Experimental Design, Materials and Methods

The soil at the experimental site is an Eutric Cambisol with 36.5 % clay, 41 % silt, and 22.5 % sand [5]. Soils were collected after harvest but before amendment addition in the autumn in order to avoid freshly added and undecomposed material. Five cores (0–20 cm, with additional depths in 1991) were collected at randomly selected locations within each plot, pooled to form a composite sample, sieved at 4 mm, air-dried and sieved at 2mm [[13], [14], [15]], yielding one sample per plot. pH was measured in a soil:water ratio of 1:2.5. Between 1956 and 1983, total soil carbon and nitrogen were analyzed using wet digestion (Walkley‐Black and Kjeldahl methods, respectively [16,17]). Subsequently both carbon and nitrogen were measured using infrared gas analysis following dry combustion (Ströhlein Instruments, Germany; LECO instruments, St. Joseph, MI). Comparison of the two methods with archived samples did not indicate the change in methods had biased soil C measurements [13,15]. Ca, P and K were measured in soils extracted with 0.1 M ammonium lactate and 0.4 M acetic acid [18]. In 1979 and 1989, K and P were also determined in 2 M hydrochloric acid soil extracts (Fig. 2). In both cases these extracts were measured using ICP-OES.

Amendment chemistry was measured for N, P, K, S and organic matter content. Total N was measured on a macro Kjeldahl apparatus. Between 1956 and 1988, P, K and S were measured on ashed samples which had been dissolved in 2 M nitric acid and diluted with water. K was measured by flame emission photometry, P was measured colorimetrically using the vanadomolybdate protocol, and S was measured gravimetrically based on barium sulfate precipitation. From 1988 K, P and S were measured using ICP-OES (ex. Perkin Elmer Plasma II [6] or Optima 3000 DV). Until 1989, amendment carbon was determined based on converting mass loss on ignition assuming a ratio of 0.5 g C g^-1^ organic matter. Organic carbon has subsequently been quantified based on infrared gas analysis following dry combustion as for the soil (Ströhlein Instruments, Germany; LECO instruments, St. Joseph, MI). Micronutrients were determined using ICP-MS (Elan 6100 ICP-MS; Perkin Elmer) following extraction with 2N nitric acid.

Aboveground crop residues (“straw”) from cereals were separated from grains, and aboveground biomass of root crops (“tops”) were separated from beets. Beets were washed prior to measurements but aboveground biomass was not. Oilseed rape was harvested as total aboveground biomass when most seeds were still green [6]. Plant C,N,K,P, S and micronutrients were measured on dried samples as described for soil amendments [7]. Crop yield was based on material dried to constant mass at 100–105 °C.

All data collected between 1956 and 2009 archived in physical binders was digitized and combined with data to be distributed as a dataset here [19]. Mass-mass plant and soil data were converted to units of mg/kg, and all yield data to kg/ha. Data were subsequently quality controlled by comparison with original data and typically observed values. Outliers, identified as single measurements from plots that were out of line with group means, were excluded. Values inconsistent with literature values were similarly removed, as were any values the initial data collectors noted were of poor quality. Redundant data were preferentially reported at the plot level (i.e. averaged to plot level when reported at subplot level and treatment level averages were excluded when plot level values were also reported). A total of 391 datapoints were removed.

## Limitations

A key limitation of the data presented here is lack of documentation on the protocols used to measure soil properties in earlier years of the experiment, as some of the people who did the work are now deceased or otherwise retired and out of contact. Soil data are missing for 2021 and 2023 because sampling shifted to every four years in 2019 and values for 2023 have not been reported yet. These data and that of subsequent years will be added to this dataset as they become available.

## Ethics Statement

The authors confirm that the current work does not include human or non-human animal subjects or data collected from social media, and that they have read to and abide by the ethical requirements for publication in Data in Brief.

## CRediT authorship contribution statement

**Grace Pold:** Writing – original draft, Writing – review & editing, Visualization. **Emme MacDonald:** Validation, Investigation, Writing – review & editing. **Sabina Braun:** Validation, Investigation, Writing – review & editing, Supervision, Project administration. **Anke M. Herrmann:** Funding acquisition.

## Data Availability

SNDSLU long-term field experiments: The frame trial (R3-RAM56), crop and soil data from 1956 and onwards (Original data).
